# Flavour perception in Alzheimer’s disease: A systematic review of olfactory and gustatory assessment methods

**DOI:** 10.1016/j.jarlif.2025.100044

**Published:** 2025-11-29

**Authors:** Danel Ushkempirova, Louise Davis, Alan Chalmers

**Affiliations:** a Warwick Manufacturing Group, University of Warwick, Coventry, CV4 7AL, UK; b Warwick Medical School, University of Warwick, Coventry, CV4 7AL, UK

**Keywords:** Alzheimer's disease, Flavour assessment, Gustatory dysfunction, Olfactory dysfunction, Taste test, Smell test

## Abstract

Disruptions in flavour perception, due to olfactory dysfunction and gustatory dysfunction, may serve as early indicators of Alzheimer’s disease and contribute to reduced quality of life. Flavour perception is a multisensory process, yet no standardised tools currently exist to assess it comprehensively in Alzheimer’s disease. This systematic review examined current olfactory and gustatory assessments used to evaluate flavour function in individuals with Alzheimer’s. Six studies met the inclusion criteria, encompassing 471 participants, including 161 with Alzheimer’s. Olfactory function was most often assessed with the Sniffin’ Sticks test, while gustatory function was typically evaluated using Taste Strips. While olfactory dysfunction was consistently reported across studies, evidence for gustatory impairment was less uniform, reflecting methodological variability in taste assessment procedures. Only one study used a culturally adapted test, and none assessed umami. However, the small number of studies, heterogeneity in methodology, and limited cultural adaptation constrain the generalisability of these findings. These findings highlight the need for a culturally adaptable, combined flavour assessment tool that minimises cognitive and linguistic demands to support early detection and monitoring of Alzheimer’s disease.

## 1 Introduction

Disruptions in flavour perception caused by olfactory and gustatory dysfunctions can profoundly affect quality of life [1] and have been identified as potential early symptoms of various neurodegenerative conditions [2]. Previous research has suggested that such deficits are prevalent in, but not limited to, patients with Alzheimer’s disease (AD) [3].

### 1.1 Flavour

For the purpose of this systematic review, it is essential to provide a clear definition of the concept of “flavour.” Several researchers [4,5] have highlighted inconsistency regarding the term “flavour” within both the scientific community and everyday English language. The ongoing debate concerning the interchangeable use of “flavour” and “taste” [5] has led to a relatively new definition that incorporates a multisensory perspective [6]. Multi-modal flavour perception is now viewed as a complex process involving the integration of olfactory, gustatory, somatosensory, and other sensory modalities [5,7].

Scholars in neurogastronomy and gastrophysics further distinguish between retronasal and orthonasal olfaction [8]. Retronasal olfaction refers to the detection of aroma molecules from the oral cavity during food and beverage ingestion, while orthonasal olfaction pertains to the capture of odour molecules in the nasal cavity during sniffing. Research indicates that retronasal olfaction plays a predominant role in flavour perception [7].

This review adopts the following definition of flavour, provided by the International Organisation for Standardisation (ISO 5492, 1992; see [4]):

*Complex combination of the olfactory, gustatory and trigeminal sensations perceived during tasting. The flavour may be influenced by tactile, thermal, painful and/or kinaesthetic effects.*

For clarity and to provide ease of understanding, *olfactory dysfunction* in the present review is used as an umbrella term encompassing any abnormality or distortion in the perception of olfactory stimuli, ranging from, but not limited to, complete loss of smell (anosmia), reduced sensitivity (hyposmia), and distorted perception of familiar odours (parosmia). Similarly, *gustatory dysfunction* refers to any deviation from normal taste perception, including but not limited to complete loss of taste (ageusia), reduced sensitivity (hypogeusia), and distorted taste perception (parageusia).

Smell and taste are two of the five principal human senses that are deeply connected with each other and are integral to flavour perception [9]. It remains unclear whether individuals can differentiate the role of taste and smell in flavour perception and accurately report deficits. The scientific report from the first Danish flavour clinic [10] provides valuable insights into smell and taste disorders and their diagnosis. It has been observed that many patients are unaware of the nature of flavour perception disruptions, often incorrectly attributing these to taste loss. Among 515 patients, 97 % reported a loss of smell, and 82 % reported a loss of taste. However, upon careful examination, only 22 % of patients were found to suffer from ageusia, whereas 89 % showed signs of anosmia. This suggests that individuals perceive flavour as a holistic experience, rather than as separate gustatory and olfactory stimuli, to the extent that distinguishing the source of flavour perception issues becomes challenging. Furthermore, the majority of individuals may incorrectly attribute these disruptions to taste loss when, in many cases, the underlying issue is anosmia. Consequently, the critical role of smell in flavour perception often goes unrecognised.

### 1.2 Flavour processing

To understand the role of olfactory and gustatory stimuli in flavour processing, it is critical to examine the brain mechanisms underlying flavour perception. Previous studies suggest that the insula, orbitofrontal cortex (OFC), and anterior cingulate cortex (ACC) are the primary regions involved in this process [11]. The degree of activation in these regions depends on familiarity with specific taste-smell combinations and the method of olfactory stimulation (orthonasal or retronasal).

A review of imaging data by [6] further explored the bottom-up processes of flavour perception, identifying the anterior ventral insula as the origin of this process, with projections to the OFC, ACC, and amygdala. This connectivity may explain the link between olfactory and gustatory dysfunction and neurodegenerative diseases, possibly due to neuronal deterioration. However, the exact mechanisms behind such dysfunctions remain unclear.

### 1.3 Assessment of flavour function

At present, there is no universally accepted measure of flavour perception, particularly in the context of neurodegenerative conditions. The relatively recent adoption of a multisensory perspective on flavour has left a gap in well-established assessment tools for clinical populations, although novel flavour-perception devices are being developed [12]. Given that taste and smell are the primary components of flavour perception, it could be argued that olfactory dysfunction and gustatory dysfunction is sufficient in this context. While secondary sensory inputs and environmental cues significantly contribute to flavour processing, their assessment becomes less critical when fundamental olfactory and gustatory functions are impaired.

Olfactory perception operates through two sensory pathways: *orthonasal olfaction*, which detects odours from the external environment, and *retronasal olfaction*, which processes odours originating within the mouth, particularly during eating or drinking [8]. Most olfactory tests focus on orthonasal olfaction and evaluate three main aspects: odour detection threshold, odour discrimination, and odour identification [13]. A widely used psychophysical instrument is the “Sniffin’ Sticks” test [14,15], which assesses these three domains collectively, referred to as the TDI score: T (Threshold) measures the lowest detectable concentration of an odour using a staircase method; D (Discrimination) evaluates the ability to differentiate between odours; and I (Identification) involves a cognitive component requiring verbal identification of familiar scents. The test employs reusable felt-tip markers infused with odours such as peppermint, cinnamon, orange, coffee, fish, and others, with the number of markers varying based on the specific test version.

In contrast, gustatory function assessment is more constrained due to the limited number of basic tastes: sweet, salty, bitter, sour, and umami (often represented by monosodium glutamate, associated with savouriness). One commonly used measure of gustatory function is the Taste Strip Test, which uses filter paper impregnated with tastants. These strips are placed on the tongue, and participants are asked to identify the taste from the basic categories [16,17].

### 1.4 Flavour and Alzheimer’s disease

A recent review examining the relationship between sensory decline and Alzheimer’s disease offers an in-depth analysis of olfactory and gustatory dysfunction, as well as the neurological mechanisms underlying these impairments [1]. Such sensory deficits not only diminish quality of life [1], but also pose significant safety risks, including an inability to detect gas leaks, spoiled food, or smoke. These risks are especially concerning for individuals predisposed to sensory impairments due to medical conditions, many of whom are not routinely screened during standard medical evaluations. The review further identified olfactory dysfunction as a promising early biomarker of AD and noted that gustatory dysfunction may also serve this role, particularly when present alongside other sensory deficits [1].

There is growing recognition of olfactory dysfunction as an emerging early biomarker of AD [3]. Evidence from a large cross-sectional study with additional longitudinal follow-up using the University of Pennsylvania Smell Identification Test (UPSIT) demonstrated a strong correlation between deficits in odour identification and AD status, and showed potential to predict progression from amnestic mild cognitive impairment (aMCI) to AD. These findings suggest that smell identification may serve as a practical and cost-effective screening biomarker, with sensitivity and specificity comparable to other established markers [18]. Previous research has also highlighted specific odours with which patients with AD experience difficulty, including plant odours (e.g., wood; [3]), sweet and fruity odours (e.g., chocolate, rose, pineapple, lemon; [19]), and culturally relevant odours (e.g., Indian ink, curry; [20]). Moreover, fMRI studies have revealed significant differences in brain activation and respiratory response patterns to unpleasant odours (e.g., pyridine, a fish-like odour), which may further distinguish individuals with AD from healthy controls [21].

In contrast, assessing gustatory dysfunction presents greater challenges, as it is difficult to isolate from other sensory modalities, contributing to the smaller body of literature and less research attention compared with olfactory dysfunction [1]. This complexity has hindered a clear understanding of gustatory decline in the Alzheimer’s disease population [1]. Nonetheless, studies indicate selective taste impairments in AD, including significantly higher detection thresholds for sweet, salty, and bitter tastes, as well as higher recognition thresholds for sweet and sour, in early-stage AD [22]. Individuals at familial risk of AD have also demonstrated poorer performance on umami recognition tests (measured using glutamic acid) after 18 months compared with matched controls, further suggesting a link between gustatory dysfunction and prodromal cognitive impairment [23]. In addition, patients with AD and mild cognitive impairment (MCI) reported significantly lower recognition of umami (measured using monosodium l-glutamate; MSG), with many unable to detect it even at the highest concentrations. This finding suggests that umami perception may decline early in the disease process, potentially reducing meal enjoyment and negatively influencing nutritional intake [24].

Several olfactory tests, including Sniffin’ Sticks [14], as well as gustatory tests such as the Taste Strips [16], have been employed in studies with AD patients. In terms of validity, Sniffin’ Sticks, Taste Strips, and the OSIT-J have each been validated in older adult populations. Sniffin’ Sticks were validated in a large German cohort and provided with age-specific normative data and diagnostic cut-off points for olfactory dysfunction, including among older adults aged 61–91 years (*N* = 350; [25]). Taste Strips were validated in a European sample from Germany, Sweden, Austria, and Switzerland (*N* = 106), with a focus on adults aged 60 years and older, and the study reported normative values alongside a 10th percentile cut-off for defining hypogeusia [17]. The OSIT-J was validated in the Japanese population across 10-year age groups, including 97 adults aged 60 years and older [26]. Although this study did not provide normative data or diagnostic cut-off points, the OSIT-J was validated against other olfactory measures, and the authors noted that its application outside Japan should be approached with caution due to potential cultural bias related to certain odours. Despite these efforts, there is currently no standardised tool designed to assess both olfactory and gustatory functions specifically for the AD population.

To address these challenges, this review introduces the novel concept of examining olfactory and gustatory impairments collectively under the term “flavour dysfunction.” This holistic perspective encourages research that overcomes the challenges of isolating individual sensory contributions by focusing instead on multisensory flavour perception to advance understanding in the field. Taken together, examining flavour perception as a whole offers a more practical and effective strategy for early diagnosis, especially given the urgent need for non-invasive, routine assessments that can monitor sensory decline in individuals at risk of Alzheimer’s disease and potentially enable earlier detection.

While assessing flavour dysfunction holds promise for understanding sensory decline in Alzheimer’s disease and may lay the groundwork for future early detection tools, the existing literature remains limited. Therefore, this review concentrates on the two primary modalities of flavour perception, olfaction and gustation, to address this notable gap. Establishing a comprehensive understanding of the existing methods for assessing olfactory and gustatory dysfunctions is essential. This systematic review aims to fill this gap by examining studies that assess both olfactory and gustatory function in Alzheimer’s patients, analysing how many studies evaluate these senses simultaneously, identifying the tests used to measure such dysfunctions, and providing an overview of flavour function in Alzheimer’s disease as a whole. Ultimately, this systematic review provides a foundation for future research and the development of standardised tools for flavour perception assessment in this context.

## 2 Methods

This systematic review aims to answer the following research question: “What are the state-of-the-art olfactory and gustatory tests used to assess flavour function in Alzheimer’s disease patients?” The question was developed using the PICo strategy (Population, Interest, Context [27]). The review protocol was retrospectively registered with PROSPERO (CRD420251135828) and reported in accordance with the PRISMA checklist to ensure transparency and adherence to systematic review reporting standards.

### 2.1 Search strategy

RQ What are the state-of-the-art olfactory and gustatory tests used to assess flavour function in Alzheimer’s disease patients?

Between February 2 and March 13, 2024, and an additional search on May 22, 2025, the following databases were searched: MEDLINE, Embase (via Ovid), PubMed, Web of Science and PsycInfo. Medical Subject Headings (MeSH) and free terms related to flavour (smell and taste), disease (i.e., AD), were used in the search. The complete search protocol can be found in Table 1, which outlines all the terms used. Terms in Table 1 were used in the following combination: ((#1 AND #2 AND #3) NOT #4). After that, all articles were extracted to a Rayyan Systematic Review Management Platform [28] for further analysis of suitability based on the eligibility criteria outlined below.

**Table 1:** Search protocol for systematic literature review.

Disease	Smell	Taste	NOT
Alzheimer*	Olfact* Smell* Perfum* Odo?r Arom* Hyposmi* Anosmi* Hyperosmi* Dysosmi*	Gustat* Tast* Flavo?r Ageusi* Dysgeusi* Hypergeusi* Hypogeusi*	Animal Review “Post-mortem” Autops* Dissection Necropsy Covid* Coronavirus Mice Mouse Rat

Terms were combined as follows: ((#1 AND #2 AND #3) NOT #4).

### 2.2 Eligibility

Based on the principles of a good-quality systematic literature review [27], the following inclusion and exclusion criteria were developed:

• Publication year (from 2000 to 2025) must capture the latest state-of-the-art of assessment tools used in AD populations to test flavour function. Therefore, all articles published before 2000 were excluded.
• The review focuses on adults with AD diagnosed using recognised diagnostic criteria. Therefore, studies without AD patients were excluded.
• Studies must either employ and report assessment methods of flavour dysfunction, specifying the tools or tests used, or employ and report both olfactory and gustatory tests, specifying the tools or tests used, in adults with AD.
• Only English-language original research articles were considered. Review papers, books, book chapters, letters, correspondence, and unpublished theses were excluded.
• Primary focus should be on assessment of flavour function in AD, and papers with a focus on other conditions (Parkinson’s Disease, COVID-19, stroke, anorexia nervosa, etc.) were excluded. An assessment of COVID-19 influences in any settings were excluded since it might affect olfactory and gustatory abilities, which could not be then attributed to the condition per se. Moreover, animal studies and post-mortem studies were excluded.

### 2.3 Study selection and screening

**Fig. 1:**
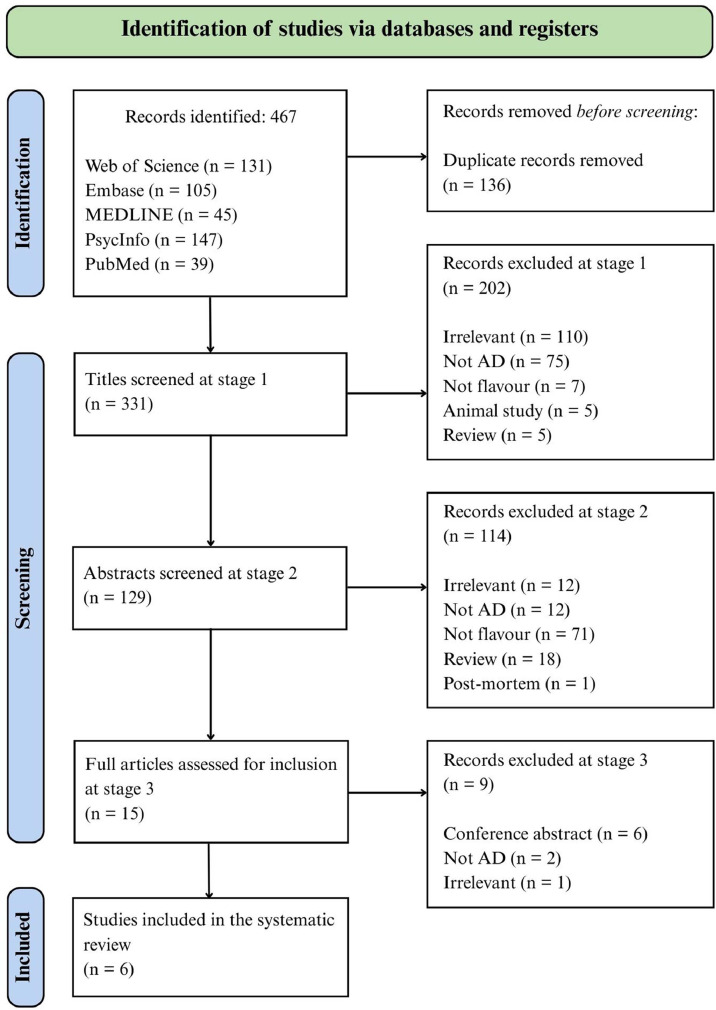
Flow diagram of study selection.

The search aimed to find tests, tools and devices, specifically used to assess Olfactory Dysfunction (OD) and Gustatory Dysfunction (GD) in AD patients. The data extraction was conducted in three phases (please see Fig. 1):

• Titles of the articles were carefully examined, and those that clearly violated eligibility criteria were excluded in phase I.
• Abstracts of the remaining articles were examined against the inclusion criteria, and all non-fitting articles were removed in phase II.
• After phases I and II, full texts of all remaining articles were examined, and only eligible studies were included in the final analysis.

Two reviewers (D.U. and L.D.) independently assessed the suitability of articles. The three-phase screening method resulted in the final list of articles relevant to the research question. Decision conflicts were resolved after the completion of each phase by both reviewers through discussion until an agreement was achieved.

### 2.4 Quality and risk of bias assessment

The quality of the included studies was assessed using the Joanna Briggs Institute Critical Appraisal Checklist for Diagnostic Test Accuracy (JBI DTA), which evaluates bias in patient selection, index tests, reference standards, and analysis [29]. Two reviewers (D.U. and A.C.) independently completed the checklist, and conflicts were resolved through discussion. Most studies reported consecutive or clearly defined samples, reducing selection bias, though some studies did not clearly describe sampling methods, resulting in uncertainty regarding representativeness. Case-control designs were generally avoided, though in some studies it was unclear whether participants were pre-selected based on disease status, which could introduce bias. Reference standards were consistently appropriate, relying on established clinical diagnostic criteria for AD. Blinding of test interpretation and pre-specified diagnostic thresholds were often unclear, which may have affected diagnostic estimates. Overall, the studies are of moderate-to-high quality, with the main sources of uncertainty stemming from incomplete reporting rather than fundamental methodological flaws.

### 2.5 Data extraction and synthesis

Data were extracted on study characteristics, participant demographics, and sensory assessment methods. Specifically, extracted information included study authors, publication year and study design, country and study centre, sample composition (e.g., number of participants with AD, mild cognitive impairment, healthy controls, or other groups), diagnostic criteria for AD, participant age, sex/gender, Mini-Mental State Examination (MMSE) scores, body mass index (BMI), and any biomarkers reported.

In particular, details of the olfactory and gustatory tests were extracted, reflecting the primary focus of the review. This included the specific tests employed, procedural details, the smells and tastes applied, and results for participants with AD, including descriptive statistics where available and an overall summary of findings.

Data extraction was performed by one reviewer and independently verified by a second to ensure accuracy and completeness. Extracted data were organised into predefined categories and tabulated, with findings narratively analysed and synthesised for each category.

## 3 Results

### 3.1 Study selection and characteristics

The Prisma diagram (see Fig. 1) outlines the review’s screening process. A total of 467 articles were identified through an initial search of Web of Science, Embase, MEDLINE, PsycInfo, and PubMed. After removing 136 duplicates, 331 titles were screened against the inclusion and exclusion criteria. In stage 2, the abstracts of 129 articles were reviewed, leading to the selection of 15 articles for full-text screening. In the final step, 6 articles met the criteria for inclusion in the analysis.

Fig. 1. For a comprehensive summary of the included studies, refer to Table 2, which outlines the study authors, publication year, location, sample size (including patient age, gender distribution, and other demographic characteristics), as well as the olfactory and gustatory tests used to evaluate sensory functions. A detailed description of the methodologies and specific tests employed is also provided in Table 3. Nearly all studies included demographically matched healthy controls, except for one [30], which compared patients with AD to those with non-Alzheimer dementia.

**Table 2:** Summary of included studies.

First Authors & Publication Year	Country	Study design	Study Centre	Sample size and diagnosis	Diagnostic criteria used	Age	Sex/gender	MMSE	BMI	Biomarkers used	Olfactory Test	Gustatory Test
Doorduijn et al. (2020)[34]	The Netherlands	Cross-sectional design	Alzheimer Center Amsterdam	92 participants: 30 CE, 22 MCI, 40 HC	NIA-AA criteria	69.5 ±9.4	53.3	24 [21- 26]	26.3 ± 4.9	CSF samples by lumbar puncture	Sniffin’ Sticks 16 & Subjective olfactory changes	Taste Strips & Preference for taste intensities
Kouzuki et al. (2018)[3]	Japan	Cross-sectional design	Tottori University	114 participants: 40 CE, 34 MCI, 40 HC	DSM-IV criteria; NINCDS- ADRDA criteria	79.5 ±1.5	42.9	20.4 ± 0.6	-	CSF samples by lumbar puncture	Odor Stick Identification Testfor Japanese (OSIT-J)	Gustatory test by the intraoral dropping method using taste solutions
Lang et al. (2006)[30]	Germany	Cross-sectional design & exploratory study	Neurological Outpatient Department, University of Erlangen	52 participants: 24 CE (5 with vascular component, 1 with Parkinson syndrome), 28 non-Alzheimer type dementia	DSM-IV criteria	-	-	-	-	-	Sniffin’ Sticks 12	Taste Strips Test & Whole Mouth Test
Petekkaya et al. (2022)[32]	Turkey	Cross-sectional design	Neurology Clinic of the Faculty of Medicine, Mustafa Kemal University	45 participants: 15 CE, 15 Parkinson’s disease, 15 HC	NINCDS- ADRDA criteria	75.40 ±8.30	53.3	18 ≤ MMSE≤ 23	-	-	Smell Questionnaire & Sniffin’ Sticks 12 & MRI Scans	Taste Questionnaire & Taste Strips & MRI scans
Schmicker al. (2023)[33]	Germany	Cross-sectional design & prospective study	German Center of Neurodegenerative Diseases (DZNE) Memory Clinic	80 participants: 18 HC, 17 SCD, 23 MCI, 22 CE	NIA-AA criteria	77.8 ±7.7	54.5	17.8 ±4.2	-	-	Sniffin’ Sticks 12	Taste Strips
Steinbach et al. (2010)[31]	Germany	Cross-sectional design	Psychiatric Department, Ludwig Maximilians University	88 participants: 29 HC, 29 MCI, 30 CE	NINCDS- ADRDA criteria	73.3 ± 7.8	56.7	21.7 ± 4.7	-	-	Sniffin’ Sticks 16	Taste Strips

Note*: Means with standard deviations (SD) are reported for age, sex/gender, MMSE, and BMI. Abbreviations: HC, Healthy Controls; AD, Alzheimer’s Disease; MCI, Mild Cognitive Impairment; SCD, Subjective Cognitive Decline; NIA-AA, National Institute on Aging–Alzheimer’s Association criteria; NINCDS-ADRDA, National Institute of Neurological and Communicative Disorders and Stroke-Alzheimer’s Disease and Related Disorders Association criteria; DSM-IV, Diagnostic and Statistical Manual of Mental Disorders, Fourth Edition; MMSE, Mini-Mental State Examination; BMI, Body Mass Index; CSF, Cerebrospinal Fluid; MRI, Magnetic Resonance Imaging.

**Table 3:** Methodological details of included studies.

First Authors &Publication Year	Olfactory Test	Procedure	Smells Used	Olfactory Test Result	Gustatory Test	Procedure	Tastes Used	Gustatory Test Result	Summary of Findings
Doorduijn et al. (2020) [34]	Burghart Sniffin’ Sticks 16 Test*^,^†; Subjective olfactory changes;	Sniffin’ Sticks 16 battery includes odour identification (ID), discrimination (DIS), and detection threshold (THR) tests. Testing was conducted with participants blindfolded. THR: 16 triplets (two odourless, one with n-butanol); threshold calculated as the mean of the final 4 out of 7 staircase reversals (range 1–16). DIS: 16 triplets with two identical and one distinct odour (range 0–16). ID: 16 pens with common odours at supra-threshold levels, 4-option multiple choice (range 0–16). TDI score: sum of ID, DIS, and THR (range 1–48). Subjective ratings: Yes/No question and 5-point scale assessing ID, DIS, and THR.	ID: Orange, leather, cinnamaldehyde, peppermint, banana, citrus, anethole, turpentine, garlic, coffee, apple, clove, pineapple, rose, fish. DIS: Target chemicals include n-octyl acetate, n-butanol, isoamyl acetate, anethole, geraniol, 2-phenylethanol, (+)-limonene, (-)-carvone, citronellal, pyridine, eugenol, eucalyptol. Non-target chemicals include cinnamaldehyde, 2-phenylethanol, anethole, eugenol, (+)-fenchone, l-menthol, linalool, α-ionon, among others. THR: Sticks 1–16 contain 2-phenylethanol or n-butanol.	TDI score for AD patients: 24.6 ± 1.3, significantly lower than controls;	Burghart Taste Strips*; Taste intensity preferences;	Taste Strips: 16 strips with varying concentrations of sweet, salty, sour, and bitter tastes were presented in randomised blocks starting from the lowest concentration. Participants placed strips on their tongues, closed their mouths, and identified tastes as sweet, salty, sour, bitter, or no taste, rinsing with water between strips. Scores ranged 0–16 (1 point per correct identification). Taste preference was assessed using lemonade and tomato juice samples with five sugar or salt concentrations, ranked 1 (least) to 5 (most liked) and rated on a 0–100 scale.	Sweet, salty, sour, and bitter; Lemonade and tomato juice with five sugar (0.0625 mol/L to 1 mol/L) or salt (0.03125 mol/L to 0.5 mol/L) concentrations;	Mean scores in AD patients: Sweet (2.9 ± 0.2), Salty (2.5 ± 0.2), Sour (1.7 ± 0.2), Bitter (1.9 ± 0.2);	MCI and AD scored lower in odour discrimination and identification compared to HC; No significant differences in olfactory threshold; No significant group differences in total gustatory scores, taste intensity, or food preferences; MCI and AD preferred sweet taste; AD patients scored significantly lower in sour taste than MCI; No differences in total taste scores.
Kouzuki et al. (2018) [3]	Odor Stick Identification Test for Japanese (OSIT-J)*†; Subjective olfactory function questionnaire;	OSIT-J: Identification only. Semi-solid cream with one of 12 familiar Japanese odours was applied to paraffin paper, rubbed to release microcapsules, and presented for identification among 6 response options. Scoring range: 0–12; participants were excluded if they detected odourless control sticks. Subjective smell function was self-rated on a 5-point scale (1 = worst, 5 = best).	Indian ink, wood, perfume, menthol, Japanese orange, curry, cooking gas, rose, Japanese cypress, fermented beans/sweaty socks, condensed milk, and roasted garlic;	For AD patients, the mean scores for olfactory function were 1.2 ± 0.2 for good odours, 1.0 ± 0.2 for plant odours, 0.6 ± 0.1 for spice odours, and 0.6 ± 0.1 for offensive odours.	Gustatory test by the intraoral dropping method* using taste solutions; Subjective gustatory function questionnaire;	Taste solutions for sweet, salty, sour, and bitter were administered intraorally via dropper starting at the lowest concentration. Participants identified tastes from six options: sweet, salty, sour, bitter, unidentifiable, or no taste. Scores ranged 1 (lowest concentration) to 6 (highest unrecognised). Participants also self-rated taste ability on a 1–5 scale (1 = worst, 5 = best).	Sweet: 0.003, 0.025, 0.1, 0.2, and 0.8 g/ml sucrose; Salty: 0.003, 0.0125, 0.05, 0.1, and 0.2 g/ml sodium chloride; Sour: 0.0002, 0.002, 0.02, 0.04, and 0.08 g/ml tartaric acid; Bitter: 0.00001, 0.0002, 0.001, 0.005, and 0.04 g/ml quinine hydrochloride;	For AD patients, the mean gustatory function scores were 2.2 ± 0.1 for sweet, 2.7 ± 0.2 for salty, 3.0 ± 0.2 for sour, and 2.3 ± 0.2 for bitter.	AD had significantly lower total OSIT-J scores compared to HC across all odour categories (e.g., good, plant, spice, offensive); No significant differences in total gustatory scores or four basic tastes among AD, MCI, and HC, though AD had the lowest mean gustatory scores; Subjective smell and taste ratings showed no significant differences between AD, MCI, and HC.
Lang et al. (2006) [30]	Sniffin’ Sticks 12 Test*^,^†	Sniffin’ Sticks 12-item identification test (8-stick version used for first two participants; scores multiplied by 1.5). Odour identification was assessed using a forced-choice paradigm with 4 response options.	Orange, leather, cinnamaldehyde, peppermint, banana, citrus, anethole, turpentine, garlic, coffee, apple, clove, pineapple, rose, fish.	No individual olfactory test results were reported for AD patients.	Whole Mouth Test and the Taste Strip Tests*	Taste assessment measured discriminative perception using a forced-choice response sheet with four pure taste qualities, minimising associative or verbal elements, which limited the separation compared to previous studies. Testing occurred in a well-ventilated room; participants could rinse with tap water as needed.	4 concentrations each of sweet (sucrose), sour (citric acid), salty (sodium chloride), and bitter (quinine hydrochloride dihydrate)	No individual gustatory test results were reported for AD patients.	No significant differences in smell or taste between Alzheimer and non-Alzheimer dementia patients.
Petekkaya et al. (2022) [32]	Sniffin’ Sticks 12 Test*^,^‡; Taste and Smell Questionnaire; MRI scans	Procedure details were not reported but likely followed the standard Sniffin’ Sticks identification test protocol. A custom Smell Questionnaire (developed by an otolaryngologist and statistician) assessed self-reported olfactory changes. 3D axial cranial MRI scans were acquired to analyse volume in brain regions associated with olfaction.	Orange, leather, cinnamaldehyde, peppermint, banana, citrus, anethole, turpentine, garlic, coffee, apple, clove, pineapple, rose, fish.	No individual olfactory test results were reported for AD patients.	Burghart Taste Strips*; Taste and Smell Questionnaire; MRI scans;	Standard Taste Strips Test procedure was likely used (no specific details reported). Participants completed a Taste Questionnaire assessing self-reported taste changes. 3D MRI scans analysed volumetric brain changes linked to taste.	4 concentrations each of sweet (sucrose), sour (citric acid), salty (sodium chloride) and bitter (quinine hydrochloride dihydrate)	No individual gustatory test results were reported for AD patients.	AD patients had significantly lower volumes in multiple brain regions related to olfactory and gustatory processing, including bilateral RO, HIPPO, and left OB; Weak negative correlations were found between left OB volume and scores on Sniffin' Sticks, Smell Questionnaire, Taste Strips, and Taste Questionnaire in AD.
Schmicker et al. (2023) [33]	Burghart Sniffin’ Sticks 12 Test*^,^†; Self-reported smell function	Burghart Sniffin’ Sticks ID-12 test assessed odour identification. Odour pens held 2 cm from the nose for 3 s and participants identified odours via 4-option multiple-choice. Max score: 12; test duration: 5 min. Self-rated smell performance was evaluated on a 0–10 Likert scale using the question: “How well would you rate your smell performance?”	Orange, leather, cinnamaldehyde, peppermint, banana, citrus, anethole, turpentine, garlic, coffee, apple, clove, pineapple, rose, fish.	No individual olfactory test results were reported for AD patients	Burghart Taste Strips*; Self-reported taste function	Taste Strips were side-separated and presented in counterbalanced ascending concentrations on alternating tongue sides, 16 strips per side covering four taste qualities at four concentrations. Participants rinsed with water between strips and identified tastes via forced choice. Maximum score: 32. Self-rated taste function on 0–10 Likert scale answering “How well would you rate your taste performance?”	4 concentrations each of sweet (sucrose), sour (citric acid), salty (sodium chloride), and bitter (quinine hydrochloride dihydrate)	AD participants had a total taste score of 15.2 (±4.3), with 7.7 (±2.4) on the left side and 7.5 (±2.3) on the right side	HC performed significantly better in smell tests than AD; No significant olfactory differences between AD and MCI; AD showed significantly lower taste scores compared to HC; No significant gustatory differences between AD and MCI;
Steinbach et al. (2010) [31]	Sniffin’ Sticks 16 Test*^,^†	The Sniffin’ Sticks 16 test battery assesses odour identification (ID), discrimination (DIS), and detection threshold (THR). ID: 16 pens, 4-choice identification (0–16). DIS: 16 triplets; identify the distinct odour in triplets (3-alternative forced choice; 0–16). THR: 16 dilutions, staircase method to detect odour (1–16). Total TDI score = ID + DIS + THR (1–48). Participants rated taste, smell, and other symptoms on a 0–100 Likert scale. Testing was done before breakfast; duration 30–40 min..	ID: Orange oil, leather fragrance, cinnamaldehyde, peppermint, banana, citrus, anethole, turpentine, garlic, coffee, apple, clove, pineapple, rose, fish. DIS: Target chemicals include n-octyl acetate, n-butanol, isoamyl acetate, anethole, geraniol, 2-phenylethanol, (+)-limonene, (-)-carvone, citronellal, pyridine, eugenol, eucalyptol; Non-target chemicals include cinnamaldehyde, 2-phenylethanol, anethole, eugenol, (+)-fenchone, l-menthol, linalool, α-ionon, among others. THR: Sticks 1–16 contain 2-phenylethanol or n-butanol.	Olfactory function mean ID (7.7 ± 3.3), DIS (8.8 ± 2.1), THR (4.5 ± 2.7), TDI (21.0 ± 6.8);	Taste Strips Test*	Taste Strips were presented in random order with increasing concentrations and 30-second intervals. Participants identified sweet, sour, salty, and bitter tastes via forced choice. Side-separated testing required participants to keep their tongue out. Maximum score: 16 (4 per taste). Participants rated taste, smell, and other symptoms on a 0–100 Likert scale. Testing occurred before breakfast; duration 6–8 min.	Taste strips contain four concentrations for each taste: sweet (0.05, 0.1, 0.2, 0.4 g/ml sucrose), sour (0.05, 0.09, 0.165, 0.3 g/ml citric acid), salty (0.016, 0.04, 0.1, 0.25 g/ml sodium chloride), and bitter (0.0004, 0.0009, 0.0024, 0.006 g/ml quinine hydrochloride);	AD participants had a total taste score of 15.2 (±4.3), with 6.2 ± 3.5 on the left side and 6.7 ± 3.1 on the right side;	Olfactory function (ID, DIS, TDI) was significantly lower in AD versus MCI and HC; THR significantly differed only between HC and AD. AD and MCI showed no significant differences in taste scores; AD patients rated subjective smell and taste significantly lower than HC; No significant differences between AD and MCI.

This table provides an in-depth overview of the experimental methodologies, including the specific tests and protocols employed to assess olfactory and gustatory function in the included studies. Please note that cultural validation markers are applied only to olfactory measures, as these typically involve odourants whose familiarity may vary substantially across cultures.

⁎ indicate tools validated in older adults [17,25,26,44]. Olfactory tests marked.

† indicates olfactory tests that have been validated in the study’s cultural or geographical population [25, 26, 43].

‡ indicate culturally modified versions validated for that population [25,26,39,43]. Please note that cultural validation markers are applied only to olfactory measures, as these typically involve odourants whose familiarity may vary substantially across cultures.

A total of 471 participants were included across the studies, comprising 161 patients with AD. Three studies applied the National Institute of Neurological and Communicative Disorders and Stroke and the Alzheimer’s Disease and Related Disorders Association (NINCDS-ADRDA) criteria [3,31,32], two used the National Institute on Aging–Alzheimer’s Association (NIA-AA) criteria [33,34], one relied solely on Diagnostic and Statistical Manual of Mental Disorders, 4th edition (DSM-IV) criteria [30], and one combined DSM-IV with NINCDS-ADRDA criteria [3]. Three studies were conducted within the last five years [[32], [33], [34]], one within seven years [3], and two within the past two decades [30,31].

Two studies validated olfactory and gustatory tests against AD biomarkers, such as cerebrospinal fluid (CSF) levels [3,34]. Only one study reported patients’ body mass index (BMI) [34].

### 3.2 Gustatory assessment in Alzheimer’s disease

The most frequently utilised measure for evaluating gustatory deficits in individuals with AD was the Taste Strips Test, employed in five studies [[30], [31], [32], [33], [34]]. The remaining study used the intraoral dropping method with taste solutions [3]. Alongside psychophysical measures, three studies incorporated subjective assessments of gustatory function [3,32,33], one included MRI analysis [32], one utilised the Whole-Mouth Test in conjunction with Taste Strips [30], and one examined taste intensity preferences [34]. One study reported a sensitivity of 60 % and specificity of 70 % based on ROC analysis for differentiating between AD patients and healthy controls, and a sensitivity of 75.0 % and specificity of 35.3 % for distinguishing between AD and MCI patients using the intraoral dropping method with taste solutions [3].

Four studies reported clearly defined methodologies, facilitating comparisons and ensuring adherence to procedural standards [3,31,33,34]. Two studies assessed side separated gustatory function [31,33], while three focused on whole-mouth gustation [3,30,34], though methodological clarity varied [30].

The studies on correlations between gustatory measures and other relevant variables in AD patients presented mixed results. One study reported no significant correlations between MMSE and total or individual taste scores, subjective assessments, or ApoE *ε*4 genotype in AD patients [31], whereas another study found significant correlations between the total gustatory test score and MMSE scores, as well as between salty taste scores and MMSE, A*β*42 levels, and the p-tau181/A*β*42 ratio [3]. Weak negative correlations between olfactory bulb volume and taste test scores were also reported [32]. Subjective taste assessments were not correlated with psychophysical taste measures in AD patients [31].

### 3.3 Gustatory function in Alzheimer’s disease

Four studies confirmed deteriorated gustatory function in AD patients compared to healthy controls [[30], [31], [32], [33]], while three others reported no significant differences between the two groups [3,30,34]. Notably, one study is listed in both groups, as its findings indicated significantly lower Whole-Mouth Test (WMT) scores in AD patients than in controls, but no significant differences with the Taste Strips Test [30].

Descriptive statistics for gustatory function in AD patients, including scores for individual tastes, were reported in three out of six studies [3,31,34]. One study provided total scores only, including left- and right-side distinctions for the tongue [33], while two studies did not report separate scores for AD patients [30,32].

Subjective measures of gustatory function were inconsistent. Two studies reported significantly worse subjective gustatory function in AD patients compared to controls [31,32], while one found no significant differences [3]. Another study identified significantly reduced sour taste perception in AD patients compared to MCI patients, though not controls, alongside a non-significant preference for sweet tastes in both AD and MCI groups [34]. Furthermore, one study linked gustatory dysfunction in AD patients to volumetric changes in specific brain regions [32].

### 3.4 Olfactory assessment in Alzheimer’s disease

Olfactory function was evaluated using Sniffin’ Sticks in five studies: three used the 12-item version [30,32,33], and two employed the 16-item version [31,34]. One study utilised the Odour Stick Identification Test for Japanese (OSIT-J) [3]. Four studies focused exclusively on the odour identification component of olfactory function [3,30,32,33], while two conducted comprehensive assessments, including odour identification, odour discrimination, and detection thresholds [31,34]. In addition to psychophysical tests, three studies incorporated custom subjective olfactory questionnaires [3,33,34], and one study included MRI analysis to investigate structural correlations [32].

Four studies provided detailed descriptions of test administration procedures, ensuring adherence to standardised protocols [3,31,33,34]. Only one study explicitly listed the scents used [3]. This study employed the OSIT-J, which was specifically adapted to include odours familiar to the Japanese population. It also reported sensitivity and specificity scores from receiver operating characteristic (ROC) analysis: distinguishing AD from controls with 82.5 % sensitivity and 67.5 % specificity and differentiating AD from MCI with 75.0 % sensitivity and 50.0 % specificity.

Correlations between olfactory function and cognitive measures in AD patients also showed mixed results. Some studies reported significant correlations between olfactory test scores and cognitive assessments such as the MMSE [3], while others found no significant associations [33]. One study reported a significant association between odour identification and total TDI score with subjective olfactory assessment [31]. Regarding AD biomarkers, no correlation was found between biomarker levels and olfaction [34]. In AD, weak negative correlations were observed between left olfactory bulb volume and Sniffin’ Sticks Test and Smell Questionnaire scores [32].

### 3.5 Olfactory function in Alzheimer’s disease

All studies reviewed confirmed diminished olfactory function in AD patients compared to healthy controls [3,[30], [31], [32], [33], [34]].

Descriptive statistics for olfactory function, including subscale scores where applicable, were reported in three out of six studies [3,31,34]. The remaining studies did not provide separate scores for AD patients, limiting direct comparisons [30,32,33]. Amongst the four studies that included patients with MCI, one found no significant differences in olfactory function between MCI and AD patients [33]. However, three studies identified significant differences [3,31,34], though with some limitations. Two studies found significant differences in odour identification and discrimination, but not in detection thresholds [31,34], while another confirmed differences specifically for plant odours [3].

Additionally, one study reported a discrepancy between subjective and psychophysical assessments: AD and MCI patients rated their olfactory function subjectively higher than what was measured by objective tests [31]. Furthermore, only one study analysed aromas by specific profiles, including pleasant odours, plant odours, spicy odours, and offensive odours [3].

### 3.6 Flavour function in Alzheimer’s disease

Assessment of flavour function may serve as a promising predictive marker for early AD, given the evidence of olfactory and gustatory dysfunction in AD. While olfactory deficits are consistently reported across studies, findings on gustatory impairments are mixed, with some studies identifying significant deficits and others finding no notable differences compared to controls [30,32]. Notably, one study observed a correlation between olfactory and gustatory function test results [3]. These findings highlight the complexity of flavour function changes in AD and emphasise the need for standardised, longitudinal research to better understand flavour dysfunction progression and diagnostic potential.

## 4 Discussion

This systematic review summarises the most commonly used measures of olfactory and gustatory function in AD patients, highlighting the insights they provide into flavour dysfunction in this population.

The Sniffin’ Sticks Test [15] was the most frequently employed measure of olfactory function, with studies using either a brief screening version assessing odour identification only, or a more comprehensive format that also evaluates odour discrimination and detection thresholds. Findings indicated significant differences between AD patients and healthy controls in odour identification and discrimination, but not in detection thresholds [31,34]. This suggests that impairments in odour discrimination and identification may be driven by memory deficits, reflecting broader cognitive impairments rather than AD-specific pathology [34]. Consistent with this, prior research confirms that odour identification and discrimination rely on higher cognitive processes, which may be variably impaired depending on disease progression [35]. Nonetheless, odour identification, alongside detection thresholds, has been proposed as a promising early indicator of AD [35]. Further studies incorporating all three domains (TDI) are needed to clarify the diagnostic value of each domain, as well as the test as a whole, ideally validated against established biomarkers. Additionally, several studies incorporating self-rated assessments of olfactory and gustatory function found that patients often lacked awareness of their impairments, rating their abilities similarly to healthy controls [31]. This unawareness may be advantageous for patient testing, as it reduces bias and anxiety during the procedure [31].

Only a subset of studies investigated the diagnostic potential of olfactory and gustatory tests [3,31,33]. The remaining studies did not report receiver operating characteristic (ROC) curve analyses, limiting the ability to directly compare the screening utility of different tests and cohorts. Future research should routinely incorporate ROC-based metrics, including sensitivity and specificity, to better establish the clinical applicability of these measures for early AD and MCI detection.

One of the reviewed studies employed an instrument tailored for the study population using culturally familiar odours, the OSIT-J [3]. The authors intentionally used culturally familiar odours for the Japanese population, for instance, Japanese orange, Japanese cypress, fermented beans, and others. Based on the significant correlation between CSF biomarkers and OSIT-J, the authors propose the use of the psychophysical instrument in AD diagnosis and monitoring. However, another study found no association between AD biomarkers (A*β*42, tau, and p-tau) and olfactory performance on the full Sniffin’ Sticks TDI [34], highlighting heterogeneity in outcomes, possibly due to methodological differences. Nevertheless, considering CSF alongside psychophysical measures strengthens the overall methodology by providing insight into whether observed sensory changes reflect underlying AD pathophysiology [34]

A separate study combined psychophysical testing with structural neuroimaging and olfactory bulb volumetry, demonstrating that sensory dysfunction reflects alterations in brain regions involved in olfactory and gustatory processing [32]. This multimodal approach helps localise deficits and supports the development of longitudinal imaging–psychophysics studies to determine whether regional atrophy or olfactory bulb volume loss precedes or parallels sensory decline.

In terms of gustatory function, gustatory assessment methods varied across studies, including Taste Strips with standardised concentrations [[30], [31], [32], [33], [34]], and the intraoral drop method [3], reflecting differences in procedure and administration. Taste Strips [16,17] consist of filter paper strips impregnated with taste solutions, whereas the intraoral drop method involves placing a solution directly into the participant’s oral cavity [3]. These differences in administration may introduce additional variables, such as chemical absorbance, surface area coverage, and delivery consistency. Furthermore, sour agents differed between studies (e.g., citric acid [[30], [31], [32], [33], [34]] vs. tartaric acid [3]), and protocols varied in whether testing was conducted across the whole mouth [3,34] or lateralised to the sides of the tongue [30,31,33]. These methodological differences could contribute to the inconsistent findings regarding gustatory function in AD.

The absence of umami assessment represents a notable gap, as prior evidence indicates impairments in gustatory function can appear early in AD, with individuals at familial risk showing reduced performance on umami recognition tests [23]. Similarly, patients with AD or MCI frequently demonstrated markedly lower ability to identify umami, with many unable to perceive it even at the highest concentrations [24]. Examining umami perception in AD patients alongside olfactory testing would be an important step forward, as this could provide valuable insights into broader chemosensory perception deficiencies in the AD population. Incorporating umami (e.g., monosodium glutamate) into standard gustatory batteries could enhance construct coverage and increase the ecological validity of flavour assessments in AD patients.

Previous research has highlighted the importance of validating olfactory measures within the study population’s country due to cultural differences and varying familiarity with odours [36,37]. Half of the studies included in the current systematic review were conducted in Germany, as pointed out by Winchester and Martyn (2020), where the Sniffin’ Sticks test was developed with the intention of catering to a European sample. Nevertheless, cross-cultural equivalence without proper validation should not be assumed. For example, the original test includes the descriptor “sauerkraut,” a German term for fermented cabbage, which might not be familiar even to culturally proximate populations. A validation study in a Danish cohort [38] concluded that the original version was not effective in that population and was only applicable following cultural adaptation. The modification procedure involved assessing the familiarity of 125 different scents using a 5-point Likert scale completed by 238 Danish participants. The validation process followed the specific recommendations [15], with a threshold set at >75 % of the sample correctly identifying each scent. The modification included updating the descriptors (filler items presented alongside the target odour as four multiple-choice options) to those deemed familiar. For instance, in the original version, the correct item “Lemon” was presented alongside “Peach,” “Apple,” and “Grapefruit,” whereas in the modified version it appeared with “Curry,” “Coffee,” and “Honey.” It is worth noting that the revised set of distractors could be argued to lack similarity to the target odour, whereas the original options all fall under one logical category - fruit. Moreover, the study did not evaluate the hedonic tone of descriptors, which is recommended [15].

It was concluded that the original Sniffin’ Sticks Test was not applicable in the Danish population [38]. Given that both Danish and German olfactory profiles could be considered within a broader “European” category, this might further imply potential issues when applying the non-modified version in culturally more distinct populations. Despite this, several studies included in the current systematic review used the original version of Sniffin’ Sticks in non-German populations, such as Turkish [32] and Dutch [34], without carrying out proper validation or referencing previously validated use of the test in these groups.

Another example, a study validating and adapting Sniffin’ Sticks for the Greek population concluded that linguistic modifications (e.g., replacing “turpentine” with “ouzo”) improved odour identification results [36]. Similarly, a validation study in the Italian population confirmed the instrument’s appropriateness, despite low familiarity with the clove odourant, which was resolved through the method of exclusion [37]. Only one non-German study utilised an olfactory measure specifically tailored to the cultural context of the study population, the OSIT-J [3]. The test included odours familiar to the Japanese population, such as Japanese orange, Japanese cypress, and fermented beans.

Consideration of cultural adaptation in olfactory testing is essential, as familiarity with specific odours varies across cultural groups. Given the vast range of possible odourants, certain scents may hold cultural relevance for one population while being unfamiliar to another. For instance, a previous study validated the Sniffin’ Sticks Test and provided normative data for the Turkish population [39], but it also highlighted possible unfamiliarity with certain odours and distractors and still suggested modifications to make specific items more culturally relevant. The authors raised an interesting point regarding particular odourants: although an “apple” odourant may appear cross-culturally recognisable, different varieties of apples differ in their regional prevalence. The apple odourant used in the original test was described as resembling a Granny Smith scent, which some participants interpreted as an air freshener, as sweeter and more sugary apple varieties are more prevalent in Turkey.

Another important consideration is cross-national comparability of results, which calls for the accurate adaptation of tests, proper validation, and normative data to ensure correct classification of normosmia, hyposmia, and anosmia [38]. Therefore, there remains a need for culturally adaptable, validated olfactory tests across diverse populations, with appropriate normative data, as an essential step toward enhancing their application in early AD detection.

In terms of cultural adaptations of gustatory tests such as Taste Strips, the five primary tastes (salty, sweet, bitter, sour, and umami) are generally considered universal and are recognised by most individuals regardless of cultural background. The main difference typically relates to the inclusion or omission of umami, particularly testing in Western populations, as individuals might struggle to identify this taste due to lower familiarity [17]. An extended version of Taste Strips, incorporating four concentrations of umami, was examined in participants from Germany and Austria [40]. It was found that during free identification, umami was confused with salty taste by the majority of participants, although they were able to correctly detect it at high concentrations, similar to the other tastants.

It remains unclear whether results from gustatory tests are suitable for cross-cultural comparison. Due to differences in dietary habits, individuals from certain cultural groups might be more or less sensitive to specific tastes, which could be inaccurately interpreted as gustatory dysfunction when, in reality, it reflects typical variation within that population. For instance, a cross-cultural study of taste perception in Japanese and Thai populations reported significantly higher recognition thresholds for all five tastes in the Thai sample compared to the Japanese one [41]. The Taste Strips test has also been validated in a Portuguese population, with results showing lower recognition thresholds and a lower cut-off score for hypogeusia compared to another study that validated the test in a broader European sample. This highlights the need for cultural validation and standardisation before such tests are employed.

In the current review, two studies from non-German populations, Turkey [32] and the Netherlands [34], used the original version of Taste Strips without referencing prior validation or conducting validation themselves. Therefore, it is important to validate gustatory tests using consistent procedures across different cultural groups and to establish appropriate normative data, particularly given the growing interest in taste and flavour dysfunction as potential early markers of Alzheimer’s disease. Ensuring culturally robust and standardised assessments is essential for accurately identifying sensory changes that may signal early neurodegenerative processes, underscoring the need for emerging approaches that can support greater cultural adaptability and methodological precision. However, progress is limited by the inability to modify odourants in commercially available measures. Future research should focus on developing olfactory, gustatory, and flavour function assessment tools that are easily customisable to include culturally familiar stimuli for diverse populations.

One recent innovative method is virtual flavour simulation [12], which uses customised blends of food-safe chemicals to simulate basic tastes, mouthfeel (e.g., astringency), and aromas, creating high-fidelity simulations of real-world flavours. The authors noted the device’s potential application in healthcare settings for conducting flavour assessments in neurodegenerative conditions. Although still at an early stage, this approach offers substantial customisation options for cultural adaptability while integrating both olfactory (retronasal and orthonasal) and gustatory modalities, providing highly controlled and precise flavour experiences. Once validated across different populations, such methods could contribute to standardisation and multisensory integration in flavour assessments and support the development of culturally adaptable, combined flavour assessment tools.

Identifying early diagnostic markers for AD is crucial to enable timely interventions [33]. While current olfactory and gustatory assessments remain limited, they nonetheless represent a promising addition to routine evaluations in AD populations [30]. A key consideration is how well patients respond to these tests and what difficulties researchers encounter during administration. Overall, olfactory and gustatory tests were generally well tolerated by patients with AD, with participants rarely experiencing difficulties in understanding instructions or completing tasks [30]. In some cases, procedural adaptations were introduced to improve feasibility: patients with low MMSE scores (<15 points) were allowed assistance from a relative, who provided support but was not present during testing to avoid influencing performance [30]. Importantly, any modifications to test instructions, including the use of visual aids, should be clearly documented to ensure transparency and replicability. Advocates of sensory testing highlight certain advantages, such as the observation that gustatory tests, unlike many neuropsychological measures, are not influenced by participants’ educational background [31]. In contrast, some authors pointed out limitations, emphasising that these tests were not specifically designed for dementia populations and thus had restricted diagnostic utility [3]. Similarly, in the study incorporating MRI, participants struggled with the lengthy scanning procedure, and researchers reported difficulties with compliance during other tasks [32]. Not all studies commented on the feasibility or challenges of administration, which represents an important methodological gap for future research.

Across the included studies, several recurring methodological limitations were identified. First, incomplete reporting of participant selection, test administration, and pre-specified diagnostic thresholds introduced uncertainty in representativeness and comparability. Second, none of the included studies explicitly reported assessor blinding during smell and taste test administration. Given the direct interaction between experimenters and participants and the use of forced-choice paradigms, potential sources of bias cannot be excluded. Future studies should incorporate blinding procedures to enhance internal validity. Third, heterogeneity in gustatory and olfactory assessment methods, including differences in delivery methods, taste stimuli, and olfactory components assessed, complicates cross-study comparisons. Fourth, the predominance of European (mainly German) cohorts limits generalisability to diverse populations. Lastly, cultural adaptation of olfactory tests was rarely implemented, and umami perception was not assessed in any of the studies included in the current review, leaving gaps in comprehensive flavour evaluation. Collectively, these limitations highlight the risk of bias in certain domains and underscore the need for standardised, culturally adaptable, and fully reported methodologies in future research.

This systematic review also had some limitations. Only six studies met the inclusion and exclusion criteria, making the scope of this systematic review limited. However, it initiates a critical dialogue on the topic, and future research should address the gaps identified. Reassessing the evidence after a growth in studies on flavour function in AD patients would be particularly beneficial. Not all studies provided comprehensive data on test methodologies or results for olfactory and gustatory assessments in AD patients, which posed challenges for direct comparison. Although the study quality appraisal and PROSPERO protocol registration were conducted after data extraction, both were completed to full standards, ensuring transparency and methodological rigour. It is also worth noting that the exclusion of studies on COVID-19 was justified, as the virus can cause chemosensory impairment, particularly affecting olfaction and gustation [42].

Overall, these findings highlight variability across studies and reinforce the need for standardised, culturally adaptable testing protocols to enhance the reliability of olfactory and gustatory assessments in AD patients. Future research could benefit from longitudinal designs and the exploration of culturally tailored testing to improve diagnostic accuracy and understanding of flavour dysfunction in AD. Additionally, the development of assessment tools that minimise reliance on cognitive processes and semantic language, such as incorporating visual aids for odour identification instead of verbal cues, could be a valuable area for further investigation. In summary, olfactory and gustatory testing are very promising methods with great potential to act as early biomarkers of AD, with continued research and methodological advancements.

## 5 Conclusion

This review highlights the urgent need for a standardised, validated tool to assess flavour function in AD patients. While olfactory dysfunction, especially in smell identification, has shown promise as an early AD biomarker, current measures are often time-consuming and focus on isolated sensory modalities, which yields inconsistent results. Gustatory assessments vary widely in methodology, taste stimuli included, and test regions, contributing further to contradictory findings. The limited number of studies, predominance of European (mainly German) cohorts, and lack of cultural adaptation introduce bias and limit the generalisability of findings. Overall, heterogeneity arises from variation in olfactory components assessed, gustatory delivery methods and taste stimuli applied, cultural stimulus sets, and incomplete reporting of procedural details. Despite promising findings, the lack of standardised, culturally adaptable, and validated tools limits their current utility in clinical settings, highlighting the need for further research before they can reliably support diagnosis or monitoring of AD.

Assessing flavour as a holistic sensory experience that integrates both smell and taste could overcome these limitations. A combined flavour function test would be more time-efficient, resource-effective, and potentially more sensitive in detecting early AD-related sensory decline. Importantly, such a test should be culturally adaptable and designed to minimise reliance on complex cognitive or language abilities, making it suitable for AD patients. Emerging technologies such as virtual flavour also hold significant clinical translational potential, as it could aid in the early detection of Alzheimer’s disease by monitoring changes in flavour perception, be integrated into multimodal diagnostic frameworks to assist clinical decision-making, and provide insights into nutritional habits and needs, therefore improving patients’ quality of life.

Future research must focus on developing and validating flavour-based assessments tailored to diverse populations, identifying specific olfactory and gustatory stimuli that AD patients struggle with. This approach promises to improve early diagnosis, monitoring, and personalised care for individuals with AD. Meanwhile, several key recommendations can guide further studies in this field: 1) the inclusion of diagnostic accuracy metrics to evaluate early AD detection potential; 2) transparency in procedural protocols with explicit reporting of blinding, test administration, modifications, adaptations, and any challenges encountered when testing AD participants; 3) incorporation of validated AD biomarkers; 4) inclusion of umami in gustatory and flavour assessments; and 5) consideration of cultural aspects in study design. Implementation of these measures will strengthen methodological rigour, facilitate cross-study comparisons, and support high-quality research into olfactory dysfunction and gustatory dysfunction as early markers of Alzheimer’s disease.

## Funding

This research is funded by a Warwick Industrial Fellowship in collaboration with Hollywood Gaming.

## Data statement

Data supporting this review consist solely of material available in the published literature. No primary data were generated.

## Generative AI disclosure

The authors verify and take full responsibility for the use of generative AI in the preparation of this manuscript. Generative AI (ChatGPT, GPT-4, OpenAI, 2025) was used strictly for language editing purposes, such as spell checking and grammar refinement. No content was generated by the AI. All text was reviewed and verified by the authors to ensure factual accuracy and compliance with the journal’s plagiarism policies.

## Ethical Statement

This study is a systematic review of previously published research and therefore did not require ethical approval.

## CRediT authorship contribution statement

**Danel Ushkempirova:** Writing – review & editing, Writing – original draft, Visualization, Validation, Resources, Project administration, Methodology, Investigation, Formal analysis, Data curation, Conceptualization. **Louise Davis:** Writing – review & editing, Validation, Resources, Methodology, Investigation, Formal analysis, Conceptualization. **Alan Chalmers:** Writing – review & editing, Validation, Resources, Methodology, Investigation, Conceptualization, Supervision.

## Declaration of competing interest

The authors declare that they have no known competing financial interests or personal relationships that could have appeared to influence the work reported in this paper.
